# Controlled ovarian stimulation and progesterone supplementation affect vaginal and endometrial microbiota in IVF cycles: a pilot study

**DOI:** 10.1007/s10815-020-01878-4

**Published:** 2020-07-15

**Authors:** Andrea Carosso, Alberto Revelli, Gianluca Gennarelli, Stefano Canosa, Stefano Cosma, Fulvio Borella, Annalisa Tancredi, Carlotta Paschero, Lara Boatti, Elisa Zanotto, Francesca Sidoti, Paolo Bottino, Cristina Costa, Rossana Cavallo, Chiara Benedetto

**Affiliations:** 1 Obstetrics and Gynecology 1U, Physiopathology of Reproduction and IVF Unit, Department of Surgical Sciences, Sant Anna Hospital, University of Torino, Via Ventimiglia 1, 10126 Turin, Italy; 2 Arrow Diagnostics S.r.l., Via Francesco Rolla 26, 16152 Genoa, Italy; 3 Virology, Public Health and Pediatrics Department, University of Torino, Turin, Italy

**Keywords:** Microbiota, Vagina, Endometrium, IVF/IVF-ICSI, Controlled ovarian stimulation, Dysbiosis, Infertility, Reproductive tract bacteria, Embryo implantation, 16S ribosomal subunit, Freeze all

## Abstract

**Purpose:**

Does controlled ovarian stimulation (COS) and progesterone (P) luteal supplementation modify the vaginal and endometrial microbiota of women undergoing in vitro fertilization?

**Methods:**

Fifteen women underwent microbiota analysis at two time points: during a mock transfer performed in the luteal phase of the cycle preceding COS, and at the time of fresh embryo transfer (ET). A vaginal swab and the distal extremity of the ET catheter tip were analyzed using next-generation 16SrRNA gene sequencing. Heterogeneity of the bacterial microbiota was assessed according to both the Bray-Curtis similarity index and the Shannon diversity index.

**Results:**

*Lactobacillus* was the most prevalent genus in the vaginal samples, although its relative proportion was reduced by COS plus P supplementation (71.5 ± 40.6% vs. 61.1 ± 44.2%). In the vagina, an increase in pathogenic species was observed, involving *Prevotella* (3.5 ± 8.9% vs. 12.0 ± 19.4%), and *Escherichia coli-Shigella* spp. (1.4 ± 5.6% vs. 2.0 ± 7.8%). In the endometrium, the proportion of *Lactobacilli* slightly decreased (27.4 ± 34.5% vs. 25.0 ± 29.9%); differently, both *Prevotella* and *Atopobium* increased (3.4 ± 9.5% vs. 4.7 ± 7.4% and 0.7 ± 1.5% vs. 5.8 ± 12.0%). In both sites, biodiversity was greater after COS (*p* < 0.05), particularly in the endometrial microbiota, as confirmed by Bray-Curtis analysis of the phylogenetic distance among bacteria genera. Bray-Curtis analysis confirmed significant differences also for the paired endometrium-vagina samples at each time point.

**Conclusions:**

Our findings suggest that COS and P supplementation significantly change the composition of vaginal and endometrial microbiota. The greater instability could affect both endometrial receptivity and placentation. If our findings are confirmed, they may provide a further reason to encourage the freeze-all strategy.

## Introduction

The microbiota of the female reproductive tract has long been studied through cultivation methods to identify the microorganisms that can be isolated and to assess their impact on reproductive physiology. However, an accurate picture of the microbial diversity in this body niche was achieved only recently, following the advent of highly sensitive molecular techniques that can identify microorganisms that cannot be grown in culture [1]. Sequencing of the bacterial 16S ribosomal RNA (rRNA) gene, encoding an essential component of the ribosome, is one of the most effective techniques in this field. The rRNA gene has hypervariable regions (V1–V9) that can be used to distinguish among even extremely similar, otherwise undistinguishable, bacteria [2].

Next-generation sequencing (NGS) methods such as 16S rRNA gene sequencing can be used to better define the bacterial community physiologically residing in the female reproductive tract: mainly *Lactobacilli* but also a small proportion of *Prevotella*, *Gardnerella*, *Atopobium*, *Sneathia*, *Bifidobacterium*, *Megasphaera*, and *Anaerococcus* [3–5].

Bacteria in the vagina, endometrium, and follicular fluid are likely to affect the reproductive process, from fertilization to implantation, and from maintenance of pregnancy to microbial colonization of the newborn [6, 7]. With regard to in vitro fertilization (IVF), the presence of certain bacterial strains in the endometrium was reported to reduce the likelihood of embryo implantation and, ultimately, the pregnancy rate [8, 9].

The physiological variability of ovarian steroid circulating levels during the normal menstrual cycle was shown to induce changes in vaginal microbiota [10]. It seems likely that the progressive, supraphysiological increase in estradiol (E2) serum levels during controlled ovarian stimulation (COS), as well as the iatrogenic increase in progesterone (P) concentration resulting from luteal phase P supplementation, induces significant changes in the vaginal and possibly also in the endometrial microbiota. How these changes influence endometrial receptivity toward embryo implantation is still largely unknown. An estrogen (or progesterone)-induced alteration of the vaginal and the endometrial microbiota could add a microbiological perspective to explain why implantation rates are reportedly higher in frozen embryo-transfer (ET) cycles vs. fresh ET following COS [11–13].

The aim of this pilot study was to investigate whether COS and progesterone luteal phase supplementation in IVF patients induce changes in the vaginal and the endometrial microbiota that could potentially influence endometrial receptivity toward embryo implantation. The changes were evaluated by using both the Bray-Curtis similarity index and the Shannon index to compare microbiota heterogeneity and stability in the two sites before and after COS. Furthermore, variation in the concentration of the individual bacterial genera was assessed.

## Materials and methods

### Patients

The study was authorized by the local ethical committee (authorization no. 0092218).

Fifteen patients undergoing IVF at the University Reproductive Physiopathology Center of Sant’ Anna Hospital, Turin were selected to participate in the study. Inclusion criteria were Caucasian ethnicity, age ≤ 42 years, basal (day 3) follicle-stimulating hormone (FSH) < 15 IU/l, anti-müllerian hormone (AMH) > 0.2 ng/ml, regular menstrual cycles (25–32 days), no previous repeated implantation failure (≥ 3 ETs of good scored embryos, with no pregnancy) or recurrent miscarriage (> 3 previous first trimester miscarriages), absence of tubal or uterine pathology (e.g., hydrosalpinx, endometrial polyps, myomas), pPROM history, and gastrointestinal disease. Patients receiving antibiotic or probiotic therapy possibly interfering with endometrial and vaginal microbiota were also excluded. Patients with a recent history of cervico-vaginal infection were also excluded; cervico-vaginal swabs were taken in all patients within 6 months prior to treatment. The swabs were analyzed by sowing on CNA agar plates (nalidixic acid colistin) followed by the MALDI-TOF technique for the identification of the most common cervico-vaginal pathogens plus real-time PCR for *Chlamydia trachomatis*, *Neisseria gonorrhoeae*, *Trichomonas vaginalis*, *Mycoplasma genitalium*, *Mycoplasma hominis*, *Ureaplasma parvum*, and *Ureaplasma urealiticum*.

### Controlled ovarian stimulation and IVF

COS was accomplished using recombinant FSH (rFSH; Gonal F®; Merck, Germany); the starting dose ranged between 100 and 300 IU/l and was tailored according to antral follicle count (AFC), AMH, and body mass index (BMI, weight in kg/height in m^2^). The initial rFSH daily dose was eventually adjusted at the initial assessment of ovarian response (day 6–7 of stimulation). Pituitary suppression was achieved by administration of GnRH-antagonist cetrorelix (Cetrotide®, Merck, Germany) according to a fixed protocol, starting from day 6 of ovarian stimulation. COS was monitored by serial transvaginal ultrasound (TV-US) plus serum estradiol (E2) measurement starting on day 6–7 of COS, and then every second day until at least two dominant follicles reached 18 mm in diameter, with appropriate E2 levels. The cycle was interrupted when no more than one follicle > 10 mm in mean diameter was seen at US on day 6–7 and serum E2 was < 100 pg/ml. Final follicular maturation was triggered by injecting subcutaneously 10,000 IU hCG (Gonasi HP®, IBSA, Switzerland); TV-US-guided oocyte pick-up (OPU) was performed approximately 36–37 h later under local anesthesia (paracervical block). Classical IVF or intracytoplasmic sperm injection (ICSI) was performed according to clinical indications. After 2 days of in vitro culture, embryos were scored according to Holte et al. [14], and 1–2 embryos were transferred in utero using a Guardia ™ Access catheter (Cook Medical®, Australia) under TV-US guidance, as previously described [15]. Luteal phase supplementation was achieved by administering 600 mg/day vaginal P (Progeffik®, Effik, Belgium) for 14 days, starting the day of ET. Pregnancy was assessed by serum hCG measurement 15 days after ET, and then confirmed 2 weeks later if at least one gestational sac was visualized at TV-US.

### Microbiota analysis

Patients underwent vaginal and endometrial microbiota analysis at two time points: in the cycle preceding COS (pre-COS) and in the cycle in which COS, OPU, and ET were performed (post-COS). For the pre-COS microbiota analysis, the patients were asked to self-monitor ovulation every day at the same time (first urine of the day) from day 10 of the cycle using a commercial kit for LH peak detection in the urine (Clearblue® Ovulation Test, Swiss Precision Diagnostics, Switzerland). When the test turned positive, an outpatient appointment was scheduled 3 days later to confirm spontaneous ovulation by measuring circulating E2 and progesterone levels. On the same day, a mock ET and a vaginal swab were performed: a sterile speculum was inserted and a vaginal swab (Copan eNat® COPAN Diagnostics, USA) was taken from the posterior fornix. Immediately thereafter, ET was simulated using a Guardia® Access catheter (Cook Medical®, Australia). The catheter consists of two components: an outer guide and an inner soft catheter; first, the guide is inserted into the cervical canal, avoiding contact with the vaginal walls; the inner soft catheter is then gently inserted into the guide and advanced until reaching the endometrium inside the uterine cavity. The inner catheter is protected from contact with the vagina or the cervix by the outer guide. After retracting the catheter without exposing its inner part to the vaginal environment, the distal end of the inner part (approximately 5–10 mm) was cut using sterile scissors and immediately placed in sterile PCR tubes. To maintain the stability of bacterial DNA [16], vaginal and endometrial samples were frozen at – 80 °C until analysis. An ultrasound examination was performed at the end of the procedure to check for the presence of corpus luteum.

Post-COS microbiota analysis was performed in exactly the same way at the time of fresh ET. A vaginal swab from the posterior fornix was taken; then, ET was performed as described, with the only difference that a medium droplet containing 1–2 embryos was released about 1 cm from the uterine fundus under TV-US guidance before retracting the catheter, cutting the tip, and storing it.

### 16S ribosomal hypervariable region sequencing

Vaginal swabs and catheter tips underwent specific treatment to obtain equal volumes for DNA extraction: 8 μl of sterile deionized water was added to the catheter tip and 100 μl to the swab. The samples were then gently vortexed to release the bacteria into the solution. The first extraction step involved digestion of the bacterial cell wall by adding 1 (catheter) or 2 (swab) μl of lysis buffer (200 mM KOH, 50 mM DTT). After centrifugation, the solution was incubated at 65 °C for 10 min; 1 (catheter) or 2 (swab) μl of neutralizing buffer (0.9 M Tris-HCl, pH 8.3, 0.3 M KCl, 0.2 M HCl) was then added. The extracted DNA was then purified: 10 μl of extract from the swab and the solution from the catheter were recovered, and 20 μl of sterile deionized water and 54 μl of magnetic beads (Agencourt® AMPure XP reagent, Beckman Coulter, Thermo Fisher Scientific, USA) were added. The samples were then incubated at room temperature for 5 min to allow the magnetic beads to bind DNA and were then placed in a DynaMag® (Thermo Fisher Scientific, USA) and washed with 300 μl of 70% ethanol to eliminate the supernatant. Both steps were repeated twice. A volume of 15 μl sterile deionized water was added to recover the extracted and purified DNA; the pellet obtained after washing with ethanol was re-suspended, and the samples were inserted again in the DynaMag®; the supernatant was recovered, purified, and stored at − 80 °C until sequencing.

Next-generation sequencing (NGS) of the bacteria-specific 16S ribosome gene was performed utilizing a microbiota solution B kit—hypervariable regions V3-V4-V6 (Arrow Diagnostics S.r.l., Italy). The B kit was composed of Enzyme Mix 1 solution containing the enzyme mixture for the PCR target, Enzyme Mix 2 solution containing the enzyme mixture for the PCR index, Amp Mix V3-V6 solution of degenerated oligonucleotides for amplifying hypervariable regions V3-V4-V6 of the bacterial 16S rDNA gene, and oligonucleotide solution for indexing amplified samples with the PCR target (Index Mix). An Illumina® MiSeq ™ system platform (Illumina Inc., USA) was used for sequencing. Raw sequencing data have been uploaded to NCBI, project title: BioProject PRJNA634237.

Taxonomic assignment and bioinformatic analysis were performed using the MicrobAT® software (Microbiota Analysis Tool; SmartSeq S.r.l., Italy). In the first phase of the analysis, reads were cleaned by a dedicated algorithm to remove short, low-quality sequences. Taxonomic assignment was then made by aligning the remaining sequences with the reference database (RDP database release 11-update 5) [17]. Only sequences that met reference criteria in the alignment phase were associated by the analysis system to the species taxonomic level (minimum length of the sequence aligning with the reference sequence ≥ 80%, similarity percentage ≥ 97%). The results were filtered by elimination of contaminating genera (*Sphingomonas*, *Renibacterium*, and *Arthrobacte*r, see Table 4), which were then detected by further analysis performed using devices not in contact with biological material.

Finally, the samples were characterized according to the Shannon diversity index (SDI), and Bray-Curtis analysis of the phylogenetic distance among genera was performed pre- and post-COS for the vaginal and the endometrial sites.

### Statistics

Statistical analysis was carried out with the R software® version 3.4.2 (R Studio, USA). This version was extended with the RAM package, designed for microbial ecology studies, genomic, and metagenomic analysis; it is similar and equivalent to others (Vegan®, Ggplot2®, Labdsv®, RColorBrewer®, and Heatplus®; https://www.rstudio.com).

Quantitative variables were compared using the Wilcoxon signed-rank test. Bray-Curtis distances were compared by non-parametric analysis of similarities (ANOSIM). A *p* value was derived based on the likelihood ratio test statistic for each factor of interest. Significance was set at *p* < 0.05.

## Results

Table 1 presents the patients’ baseline characteristics; all women were spontaneously ovulating and had normal P levels in the luteal phase of the cycle preceding COS.

**Table 1:** Baseline characteristics of patients. Values are expressed as mean ± SD

Patient age (years)	35.1 ± 4.3
Partner age (years)	39.6 ± 5.4
Duration of infertility (years)	3.3 ± 1.0
BMI (kg/m^2^)	23.5 ± 4.4
Antral follicle count (AFC)	15.0 ± 4.3
AMH (ng/ml)	3.9 ± 2.4
Basal (day 3) FSH (IU/l)	6.1 ± 1.3
Estradiol (LH peak + 3 days, pre-COS; pg/ml)	135.3 ± 41.2
Progesterone (LH peak + 3 days, pre-COS; ng/ml)	11.5 ± 6.4
Sperm concentration (M/ml)	41.4 ± 25.7
Sperm progressive (A+B) motility (%)	26.0 ± 13.5
Sperm normal morphology (%)	10.1 ± 7.1

*BMI* body mass index; *AMH* anti-müllerian hormone; *FSH* follicle-stimulating hormone

Table 2 presents the outcome of the IVF cycle; the circulating levels of E2 and P at ET (post-COS) were significantly higher than the pre-COS levels (819.2 ± 187 vs. 135.3 ± 41.2 pg/ml for E2, post-COS, and pre-COS, respectively, *p* < 0.05; 32.1 ± 7.4 vs. 11.5 ± 6.4 ng/ml for P, post-COS, and pre-COS, respectively, *p* < 0.05) (Tables 1 and 2).

**Table 2:** IVF treatment outcome. Values are expressed as mean ± SD or percentage

Daily exogenous FSH dose (IU)^a^	199.1 ± 43
Total exogenous FSH dose (IU)^a^	2467.2 ± 731
Peak estradiol at trigger (pg/ml)	2218.0 ± 1143
Progesterone at trigger (ng/ml)	0.7 ± 0.8
Retrieved oocytes	9.4 ± 5.5
Mature (MII) oocytes	8.0 ± 4.6
Fertilized (2PN) oocytes	5.7 ± 4.1
Cleaving embryos	5.6 ± 3.9
Mean embryo score^b^	7.6 ± 1.0
Number of embryos transferred	1.4 ± 0.9
Endometrial thickness at ET (mm)^a^	9.7 ± 1.7
Estradiol at ET (pg/ml)	819.2 ± 187
Progesterone at ET (ng/ml)	32.1 ± 7.4
Pregnancy rate/ET	46.6% (7/15)

^a^*FSH* follicle-stimulating hormone; *ET* embryo transfer

^b^Embryo score is expressed using the score by Holte et al., 2007

**Fig. 1:**
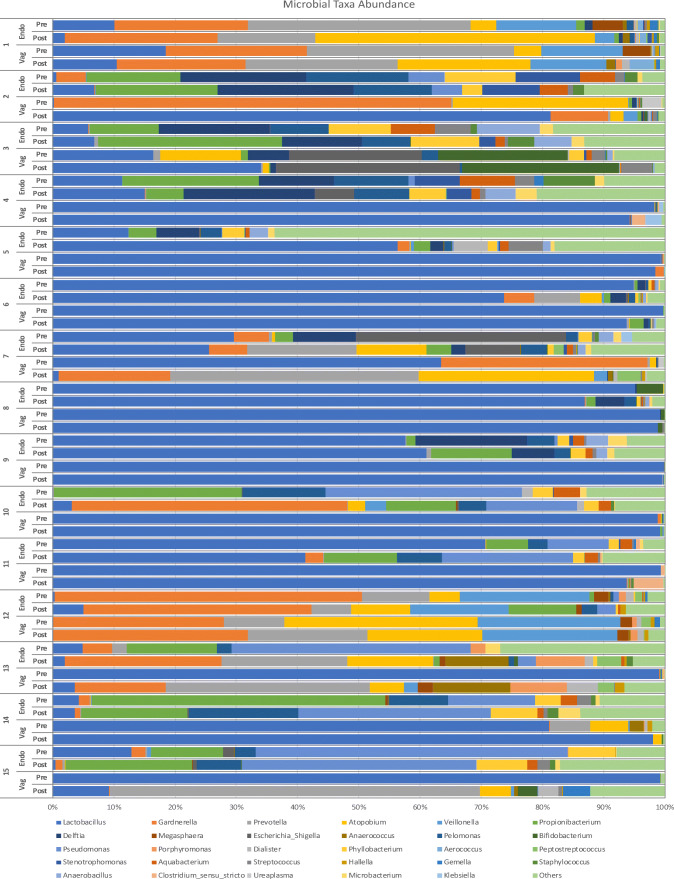
Relative abundance of the 30 most present bacterial genera in the vaginal and endometrial microbiota (pre-COS and post-COS). In each line, pre refers to pre-COS analysis, post to post-COS analysis. Other bacteria, not listed in the figure include *Finegoldia*, *Curvibacter*, *Ochrobactrum*, *Mobiluncus*, *Howardella*, *Bacteroides*, *Streptophyta*, *Peptoniphilus*, *Ruminococcus*, *Alloscardovia*, *Paracoccus*, *Anaerosphaera*, *Caulobacter*, *Kocuria*, *Serratia*, *Neisseria*, *Ralstonia*, *Parvimonas*, *Solobacterium*, *Enhydrobacter*, *Acinetobacter*, *Enterococcus*, *Rothia*, *Granulicatella*, *Corynebacterium*, *Actinomyces*, *Campylobacter*, *Fusobacterium*, *Bacillus*, *Slackia*, *Peptococcus*, *Acidaminococcus*, *Oscillibacter*, *Saccharibacteria_genera_incertae_sedis*, *Sutterella*, *Gp3*, *Cloacibacterium*, *Moryella*, *Gemmiger*, *Spartobacteria_genera_incertae_sedis*, *Paenibacillus*, *Negativicoccus*, *Propionimicrobium*, *Blautia*, *Faecalibacterium*, *Pedobacter*, *Rhodococcus*, *Meiothermus*, *Allisonella*, *Carnobacterium*, *Lachnospiracea_incertae_sedis*, *Neochlamydia*, *Diaphorobacter*, *Brevibacterium*, *Methylobacterium*, *Haemophilus*, *Micrococcus*, *Azomonas*, *Clostridium_XVIII*, *Herbaspirillum*, *Oribacterium*, *Sarcina*, *Nevskia*

A total of 81 and 90 bacterial genera were isolated from the vagina and the endometrium, respectively. Figure 1 shows the 30 most prevalent genera of the vaginal and the endometrial microbiota: In 13/15 patients (86.7%), a higher than 10% difference in the pre-COS and post-COS bacterial composition of the microbiota (relevant for optimal taxonomic unit (OTU) classification and relative abundance analysis) was detected. The pre-COS vs. post-COS difference was very marked in some samples because of the post-COS increase in potentially pathogenic genera, including *Atopobium*, *Gardnerella*, and *Pelomonas*.

Table 3 lists the ten most prevalent bacterial genera (relative abundance analysis) in the vagina and the endometrium. In the vaginal specimens, *Lactobacillus* was the most prevalent genus pre-COS and post-COS, although it was less abundant in the post-COS vaginal swabs than before COS (71.5 ± 40.6% pre-COS vs. 61.1 ± 44.2% post-COS, respectively; *p* > 0.05). An increase in pathogenic genera in the vaginal microbiota was noted for *Prevotella* (3.5 ± 8.9% pre-COS vs. 12.0 ± 19.4% post-COS, respectively; *p* > 0.05) and *Escherichia-Shigella* (1.4 ± 5.6% pre-COS vs. 2.0 ± 7.8% post-COS, respectively; *p* > 0.05) (Table 3).

**Table 3:** Proportion of the 10 most abundant bacterial genera in the vaginal and endometrial microbiota pre-COS and post-COS

Bacterial genus	Vagina pre-COS	Vagina post-COS	Endometrium pre-COS	Endometrium post-COS
*Lactobacillus*	71.5 ± 40.6	61.1 ± 44.2	27.4 ± 34.5	25.0 ± 29.9
*Gardnerella*	10.0 ± 19.2	6.5 ± 10.2	6.1 ± 13.5	10.1 ± 15.2
*Prevotella*	3.5 ± 8.9	12.0 ± 19.4	3.4 ± 9.5	4.7 ± 7.4*
*Propionibacterium*	0.1 ± 0.3	0.3 ± 0.6	11.5 ± 13.5	10.2 ± 8.9
*Pseudomonas*	0.0 ± 0.1	0.0 ± 0.1	10.3 ± 16.7	7.8 ± 12.7
*Atopobium*	5.7 ± 10.6	5.6 ± 9.4	0.7 ± 1.6	5.8 ± 12.0*
*Delftia*	0.5 ± 1.7	0.1 ± 0.3	6.0 ± 7.9	5.1 ± 7.7
*Pelomonas*	0.2 ± 0.7	0.1 ± 0.1	5.5 ± 5.4	5.4 ± 5.0
*Veillonella*	2.5 ± 6.7	2.8 ± 6.2	2.3 ± 6.2	1.6 ± 4.2
*Escherichia coli*/*Shigella spp.*	1.4 ± 5.6	2.0 ± 7.8	2.5 ± 8.8	1.1 ± 2.7

Values are expressed as percentage ± SD

^*^p < 0.05 pre-COS vs. post-COS

The endometrial microbiota was extremely heterogeneous, though *Lactobacillus* was the prevalent genus pre-COS and remained the prevalent genus post-COS (27.4 ± 34.5% and 25.0 ± 29.9%, pre-COS and post-COS, respectively; *p* > 0.05). An overall increase in the prevalence of potentially pathogenic genera was observed post-COS: *Prevotella* was significantly increased from 3.4 ± 9.5% to 4.7 ± 7.4% (*p* = 0.0494), and *Atopobium* was significantly increased from 0.7 ± 1.6% to 5.8 ± 12.0% post-COS (*p* = 0.0178).

**Fig. 2:**
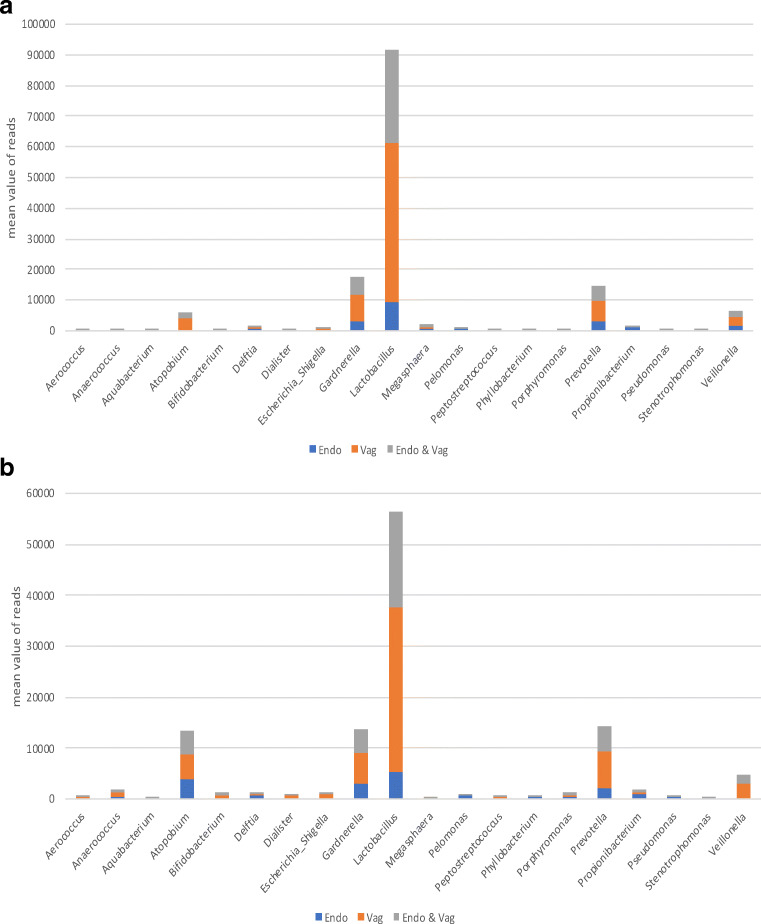
Distribution of bacterial genera in endometrial (blue part of each column), vaginal (orange part of each column), and endometrial and vaginal (gray part of each column) microbiota, pre-COS (**a**) and post-COS (**b**). Values are expressed as mean number of reads

**Fig. 3:**
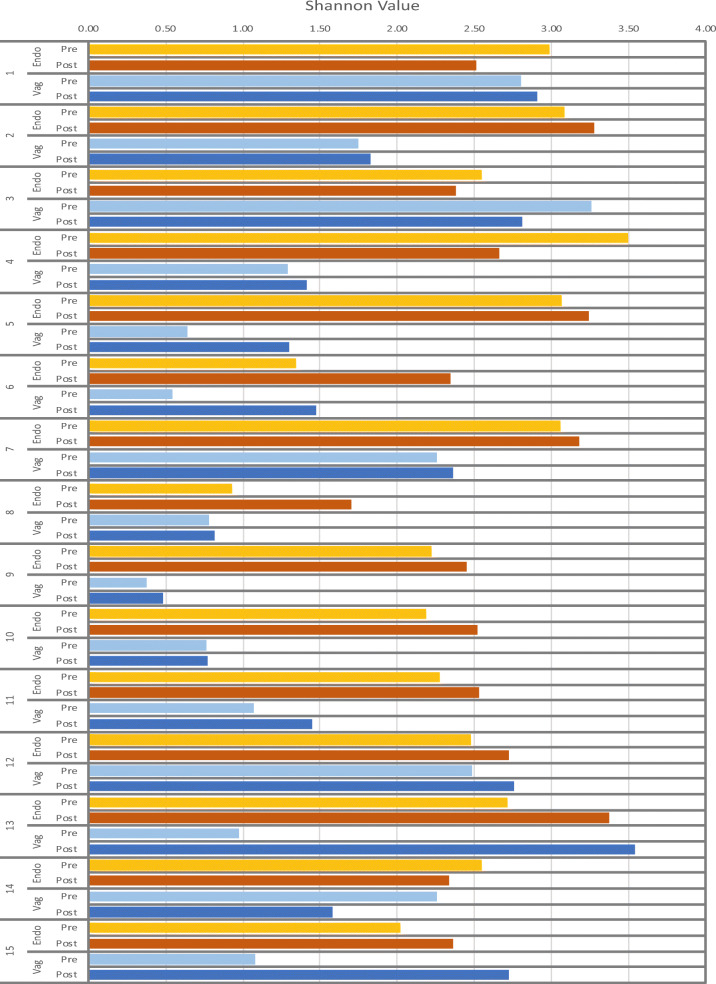
Shannon values of vaginal and endometrial microbiota, pre-COS and post-COS

**Fig. 4:**
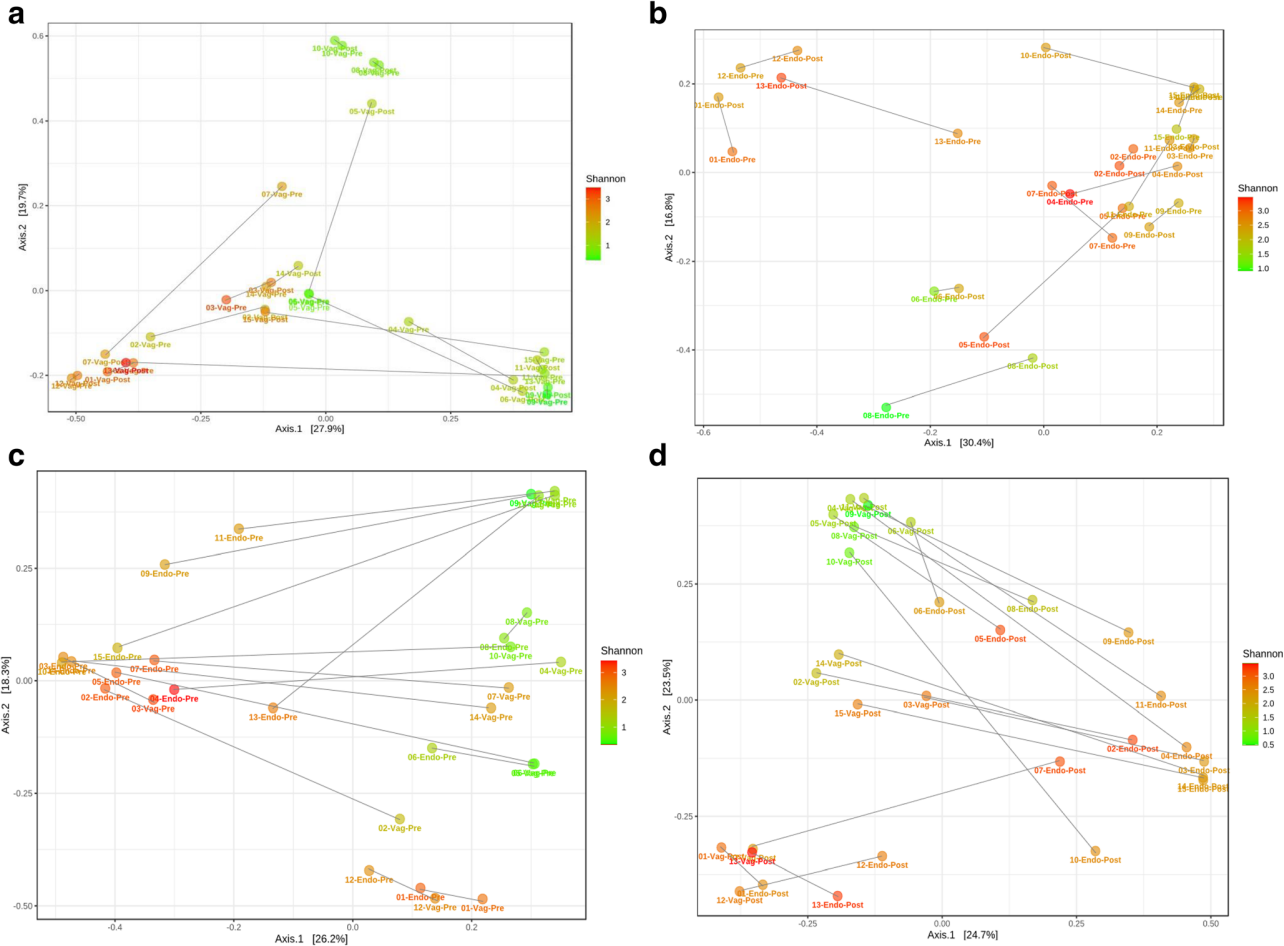
Analysis of the phylogenetic distance among bacterial genera calculated by the Bray-Curtis method for the endometrium (**a**) and for the vagina (**b**) and for the paired each type of sample, pre- and post-COS (**c**, **d**). Color data points according to alpha diversity (Shannon index). Connecting lines join the paired samples. The Bray-Curtis distance index is calculated with the formula 1 − (2*w*/*a* + *b*), where *w* is the sum of the minor score for species that are present in both communities, *a* is the sum of the taxa measures in a community, and *b* is the sum of taxa measures in the other community. The distance index was significantly increased post-COS

A comparison between the most prevalent 20 OTUs in the vaginal and the endometrial samples at pre-COS and post-COS analysis is shown in Fig. 2 a and b, respectively. The slight degree of overlap in the two microbiota (gray part of the columns) suggests a difference in the microbiota at both sampling sites between pre- and post-COS analyses. Also, the Shannon biodiversity index showed differences between pre-COS and post-COS genera at both sampling sites (Fig. 3). Biodiversity was greater in the endometrial microbiota at both time points, indicating that vaginal and endometrial microbiota had different characteristics (*p* < 0.05). In both sites, biodiversity was significantly greater after COS (*p* < 0.05), particularly in the endometrial microbiota, as confirmed by Bray-Curtis analysis of the phylogenetic distance among bacteria genera, which resulted significantly higher post-COS (Fig. 4 a and b). Bray-Curtis analysis confirmed significant differences also for the paired endometrium-vagina samples at each time point (Fig. 4 c and d).

## Discussion

Menstrual cycle phases and changes in circulating estrogen levels over a woman’s lifespan are associated with changes in the composition of vaginal microbiota [18, 19], but the effect(s) of exogenous FSH administration, with the consequent supraphysiological increase in E2, as well as P supplementation in the luteal phase, have not been studied to date.

A similar knowledge gap exists for the endometrial microbiota. Until a few years ago, the uterine cavity was believed to be sterile, and the presence of bacteria was associated with pathological colonization by vaginal bacteria; rather recently, it was demonstrated that the endometrium has its own microbiota and that the microbiological characteristics of the vagina and the endometrium only partially overlap [8].

As ovarian steroids produced during the physiological menstrual cycle affect vaginal microbiota, it seems plausible that also the uterine microbiota could be influenced by the fluctuations in E2 and P levels during cycle phases; this hypothesis has never been investigated using advanced techniques, however. Similarly, the impact of COS and P supplementation during IVF cycles on vaginal and endometrial microbiota has never been studied. Theoretically, the effects of ovarian steroids on the composition of vaginal and endometrial microbiota could be significantly amplified during COS in comparison with the physiological menstrual cycle, as COS induces wider and more rapid changes in E2 serum levels than the natural cycle, while P supplementation after ET increases circulating P far beyond physiological levels.

Based on these premises, the aim of the present study was to evaluate the impact of COS and P supplementation on vaginal and endometrial microbiota. The latter was sampled using a previously described technique that avoids contamination by vaginal flora [20]. Moreover, the vaginal and the endometrial samples were submitted to a highly sensitive, novel molecular methods (such as NGS of bacterial 16S RNA) for a thorough analysis of the bacterial genera, biodiversity, and microbial instability. Unfortunately, the high costs of the NGS technique limited the possibility of enrolling a larger patient population and to relate microbiota composition to IVF outcome, an endpoint that was beyond the scope of this study.

We report for the first time data suggesting that COS and P supplementation induce significant changes in the vaginal and the endometrial microbiota at the time of the so-called implantation window [21, 22] when fresh ET is performed. The relative proportion of vaginal and endometrial *Lactobacillus* was decreased during the IVF cycle (post-COS), with a simultaneous increase in potentially pathogenic bacteria, such as *Atopobium*, *Escherichia-Shigella*, and *Prevotella*. An unfavorable change in the vaginal and the endometrial microbiota after IVF (COS and P supplementation) occurred: the increase in the microbial instability of the uterine cavity (higher Shannon biodiversity index) was particularly pronounced.

This finding seems to contradict evidence that raising the levels of circulating estrogen induces an increase in Lactobacillus in the vagina [23, 24]. However, the estrogen levels during ART treatment exceed by an order of magnitude the natural levels during spontaneous ovulation, with completely different kinetics depending on the type of COS. The effects of these supraphysiological levels on vaginal and endometrial microbiota have never been investigated so far.

However, whether or not these changes in the endometrial microbiota might affect IVF outcome or increase the incidence of obstetric complications is still unknown. Some authors have criticized the hypothesis that endometrial microbiota plays a role in IVF outcome [25] and a recently published review [26] even questioned the existence of an active endometrial microbiota. The conclusions of the review have been challenged and critical issues raised [27], particularly as regards the variations in endometrial microbiota composition in relation to hormonal changes and compared with the vaginal microbiota. The present study provides evidence in this direction: the Shannon biodiversity index of the vaginal and the endometrial samples differed before and after COS and the biodiversity of the endometrial microbiota appeared to be greater than the vaginal microbiota. In interpreting our results, a potential contamination effect of the pre-COS on the post-COS analysis should be considered. However, the invasiveness of a vaginal swab and a transfer simulation is negligible, so much so that it is routinely performed by some IVF centers. Furthermore, a blank analysis of the devices used for the analyses was carried out to exclude possible contaminants and their impact on subsequent analysis. It is unlikely, therefore, that a minimal presence of bacteria, as identified in the blank control (Table 4), could disrupt a microbiota dominated by Lactobacilli in the time (about 1 month) between the two analyses. Another potential contamination could have been the self-administration of vaginal progesterone. Patients were advised to wear sterile gloves during administration and our analyses did not demonstrate a relevant presence of bacteria of cutaneous origin in post-COS results. For these reasons, it is reasonable to assume that the microbiota changes could be COS-related.

**Table 4:** Analysis of potential contaminants in the “white” sample and the average of the relative abundances detected in vaginal and endometrial analyses. *Sphingomonas* was excluded from vaginal and endometrial microbiota analysis because recognized by previous works as a contaminant^a^. *Arthrobacter* and *Renibacterium* are genera found in soil and water; we recognized them as contaminants^b,c^. The other genera were not excluded because they were present in negligible quantities in the blank sample and described by previous works on vaginal and endometrial microbiota

% value	Mean % value		Genus
Blank control	Endometrial catheter	Vaginal swab	
38.54	24.36	1.00	*Sphingomonas*
10.65	5.32	0.28	*Arthrobacter*
6.38	1.71	0.10	*Pseudomonas*
2.04	2.17	0.08	*Propionibacterium*
1.44	0.05	0.00	*Acinetobacter*
1.26	0.21	0.03	*Staphylococcus*
0.43	1.08	0.03	*Pelomonas*
0.25	0.24	0.01	*Renibacterium*

^a^Hashimoto T, Kyono K. Does dysbiotic endometrium affect blastocyst implantation in IVF patients? J Assist Reprod Genet. 2019;36:2471–9

^b^Koh H-W, Kang M-S, Lee K-E, Lee E-Y, Kim H, Park S-J. Arthrobacter dokdonellae sp. nov., isolated from a plant of the genus Campanula. J Microbiol. 2019;57:732–7

^c^Hirvelä-Koski V. Renibacterium salmoninarum: effect of hypochlorite treatment, and survival in water. Dis Aquat Org. 2004;59:27–33

Although *Lactobacillus* was predominant in both, the presence of other bacteria and a more heterogeneous environment compared with the vagina makes the endometrium an interesting niche to explore.

Endometrial dysbiosis and subclinical chronic endometritis have been claimed to be possible causes of repeated implantation failure or recurrent miscarriage [28–30]. Furthermore, the relative vaginal abundance of *Atopobium*, *Escherichia-Shigella*, and *Prevotella* was associated with preterm delivery [31] and a local pro-inflammatory state potentially affecting placentation [32]. Also, IVF pregnancies are known to be associated with a higher-than-normal risk of obstetrical complications, including those of infectious origin (e.g., preterm delivery, premature rupture of membranes) [33, 34].

Clarifying whether the endometrial microbiota after COS might affect IVF outcome or not will be the objective of our next study, taking into account the reportedly higher implantation rate observed by some authors when, after the so-called freeze-all strategy, thawed embryos are transferred in a cycle different from the one in which COS is performed [35]. Indeed, in a natural cycle, when E2 and P levels are physiological, the reported iatrogenic changes in pre-COS endometrial microbiota would not occur, and embryos could be transferred in a more physiological intrauterine environment.

One limitation of the present study is the small study sample size (*n* = 15). Nonetheless, analysis of four samples from the same patient, taken at two sites and at different time points in IVF treatment, makes this study one of the few with a highly detailed characterization of intra-individual variability of the microbiota of the female reproductive tract. This is a fundamental theme in the study of microbiota. Indeed, frequent fluctuations in the composition of vaginal microbiota have been documented by microscopy and cultivation studies [36–38]. Fluctuations can be triggered by genetic predisposition in different ethnic groups, menstruation or sexual behaviors, and a history of bacterial vaginosis or be driven by uncharacterized factors, especially in not Lactobacillus-dominated microbiota [39]. Although we have tried to limit these factors (Caucasian ethnicity, exclusion of patients with cervico-vaginal infections in the last 6 months), intra-individual variability in microbiota study remains a factor to be taken into account, and the results should be interpreted with caution.

Further research into the impact of the microbiota on reproduction should be focused in this direction. For example, it is necessary to integrate information on the female microbiota with that of seminal fluid, which has its own microbiota [40, 41]. Indeed, it is likely that in the future our attention will shift from studying the individual microbiota to the “reproductive microbiota” of the couple. In this view, it will be interesting to integrate the impact of the “bacterial microbiota” with that of the “virota,” which together can potentially impact IVF outcomes, as recently demonstrated [42].

Furthermore, it will be of pivotal importance to define the characteristics of the endometrial microbiota and understand its physiological alterations during the menstrual cycle. These premises are fundamental to fully understand the potential repercussions of the microbiota on implantation and also on the evolution of pregnancy. Clinicians must be aware of the fluctuations of the reproductive microbiota in order to identify the transitory conditions that can be associated with better reproductive and obstetric outcomes.

In conclusion, our pilot study shows for the first time with NGS of the 16S ribosomal subunit that COS and P supplementation routinely performed during IVF treatment induce unfavorable changes in the composition of vaginal and endometrial microbiota, increasing the proportion of potentially pathogenetic genera at both sites and significantly increasing the biodiversity and the environmental instability of the uterine cavity during the so-called implantation window.

## Data Availability

All data are available under request to the authors.
